# Ethical Issues in Photovoice Studies involving Key Populations: A Scoping Review

**DOI:** 10.1007/s41649-023-00264-3

**Published:** 2023-11-13

**Authors:** Chong Guan Ng, Sing Qin Ting, Rumana Akhter Saifi, Adeeba Bt Kamarulzaman

**Affiliations:** 1 Department of Psychological Medicine, Faculty of Medicine, University of Malaya, Kuala Lumpur, Malaysia; 2 Department of Medicine, Faculty of Medicine, University of Malaya, Kuala Lumpur, Malaysia

**Keywords:** Photovoice, Marginalized communities, HIV, Stigma

## Abstract

Photovoice, a community-based participatory research method, employs images and words to convey participants' needs, concerns, and desires. It proves particularly valuable in researching marginalized communities who face elevated health risks, disease transmission, and social and health disparities. This paper seeks to investigate the ethical considerations inherent in photovoice research projects. We conducted an extensive literature review spanning four databases to identify pertinent photovoice studies. Ethical issues from the selected articles were identified, categorized, and summarized. Our analysis of twenty-five photovoice studies uncovered various ethical concerns, which had been grouped into informed consent, participant safety and disclosure, privacy and confidentiality, misrepresentation, power dynamics, and compensation. In essence, our findings underscore the importance of addressing these ethical concerns to uphold the rights and autonomy of participants, even as photovoice research strives for authenticity, inclusivity, and empowerment.

## Background

Photovoice was first introduced in 1992 by Caroline C. Wang of the University of Michigan, and Mary Ann Burris in a project to empower the disadvantaged rural women in Yunnan Province, China, to share their voices on the policies and programs affecting them (Wang and Burris 1997). Since then, photovoice has been adopted and used in different research settings and populations worldwide. Wang and Burris defined Photovoice as a “*process by which people can identify, represent and enhance their community through a specific photographic technique*” (Wang and Burris 1997). The idea of photovoice is to make use of images and words together to express the participants’ individual or their community needs, concerns, or desires. It is a qualitative research method used mainly in community-based participatory research. In the photovoice project, participants take their own photos and use their own words to respond to an interview, share their thoughts or answer questions that are closely related to their community (Nykiforuk et al. 2011).

In this context, the designation "marginalized communities" pertains to specific demographic groups that confront an elevated susceptibility to contracting or transmitting health-related conditions, in addition to grappling with pronounced social and health disparities. On certain occasions, these groups may also be denoted as "key populations" or under other synonymous terms. Their proclivity toward vulnerability or marginalization stems from various factors, including their behaviors, occupations, socioeconomic standing, and the intricate interplay of legal and societal milieu (WHO 2011). Examples of such groups encompass men who have sex with men (MSM), people who inject drugs (PWID), sex workers, transgender individuals, and individuals living with HIV/AIDS.

Multiple discussions have taken place regarding the ethical and practical aspects of photovoice research, including subjects' recruitment, consent processes, data collection, invasion of privacy, and results dissemination, suggesting that there is a pressing need to establish a framework for monitoring the ethical considerations of photovoice research.

The aim of the review is to review the ethical challenges that may arise from a photovoice research project in general.

## Methods

### Search Procedure

A scoping review is performed to determine the ethical issues in applying the photovoice method in research. It provides a summary of findings drawn from existing literature and helps to make recommendations for future photovoice studies. A search of the literature using online electronic databases was conducted. The database for the search included PsycINFO, PubMed, Web of Science, and MEDLINE/PubMed. The keywords used in the scooping review are ((((((TI = (gender)) OR TI = (sex*)) OR TI = (drug)) OR TI = (substance)) OR TI = (HIV))) AND TS = (photovoice).

### Inclusion/Exclusion Criteria

The title and abstract of the articles were screened following the inclusion criteria: (1) original research published in peer-reviewed academic journals, (2) being in the press before May 2023, (3) primary studies that apply Photovoice as the research method, (4) studies involved marginalized communities and/or key populations, and (5) being written in English.

Articles were excluded based on the following criteria: (1) review articles, reports, opinions, educational materials, health promotional articles, methods papers, presentations, and theoretical papers, (2) duplicate publications, and (3) non-English literature.

### Screening and Results Charting

Extracted titles and abstracts from the search were reviewed by the first and second authors. The two reviewers met to perform the abstract review process and reached a consensus regarding article selection for further data extraction. The information was extracted and tabulated using a data extraction form in Excel. The following categories of information for each study were extracted: (1) authors, (2) aims of the study, (3) study designs/ methods, and (4) ethical issues related to the studies.

## Results

**Fig. 1:**
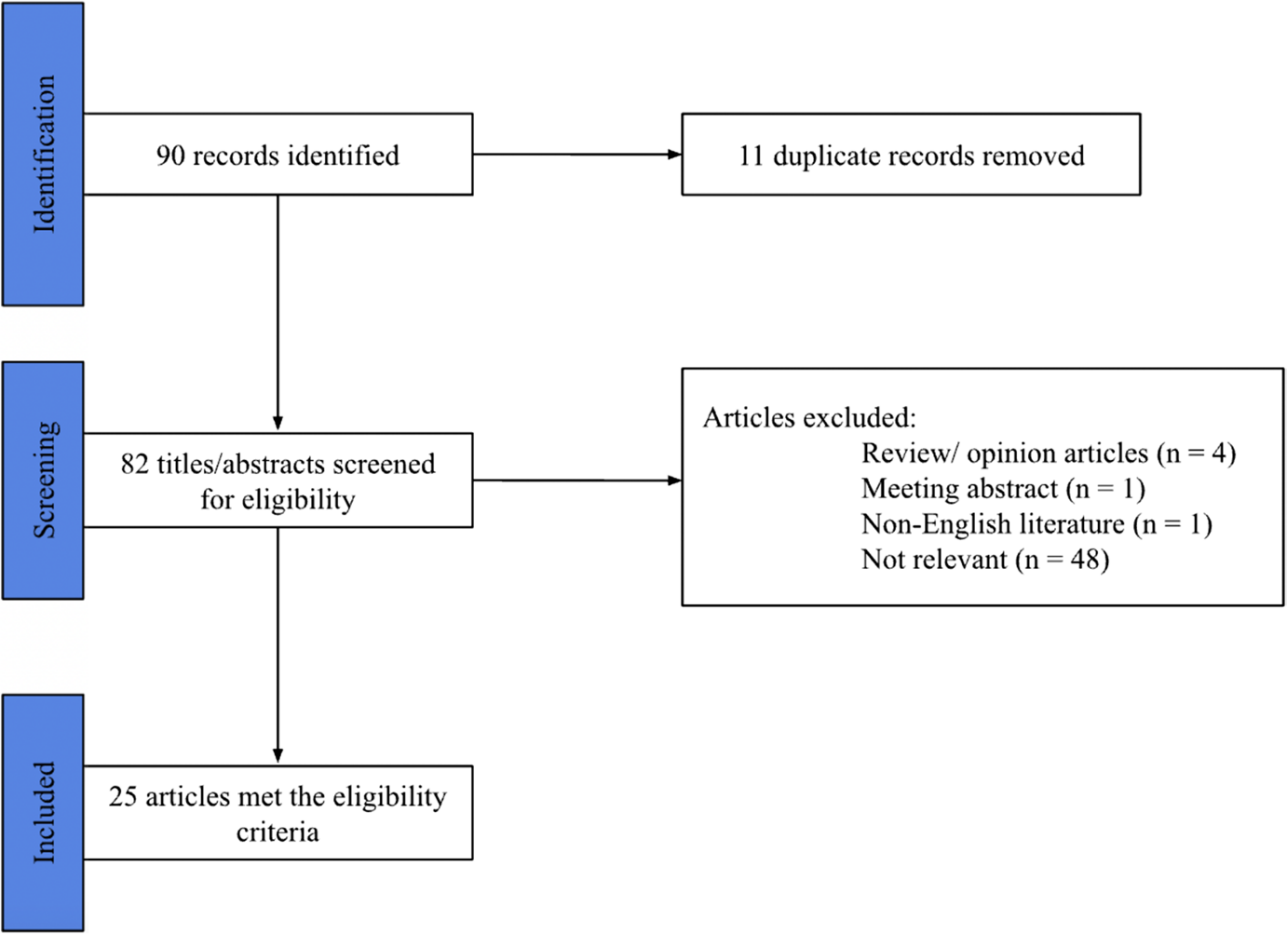
Screening and selection process for the review, PRISMA flowchart for this study

Out of the initial 90 abstracts identified, a total of 11 duplicate abstracts were excluded from consideration. Subsequently, the remaining 82 abstracts underwent screening, resulting in the inclusion of 25 abstracts for further analysis (Fig. 1).

The reasons for excluding the 57 articles were as follows: 4 articles were identified as review or opinion articles, 1 article was not written in English, 1 article was a meeting abstract, and 48 articles were deemed not relevant to the study.

Among the 25 articles included in this study, 5 of them focused on drug use, 9 explored the experiences of patients living with HIV, 5 centered around individuals living with HIV in general, 4 delved into the experiences of gender identity patients, and 2 examined the perspectives of sex workers (Table 1).

**Table 1:** Description of the included studies

No	Authors	Aims	Study design/ method	Ethical issues
	***People Who Use Drug (PWUD)***			
1	Padilla et al. (2018)	Connect PhotoVoice with the theoretical orientation of critical medical anthropology to identify local interpretations of complex social and structural factors that are most salient to the well-being of local Dominican populations affected by drug addiction	PhotoVoice 7 street-based PWUD in Santo Domingo	Privacy and Confidentiality: The participants were taught on photographing ethics
2	Valdez et al. (2019)	Use Youth Participatory Action Research (YPAR) methods and Photovoice to identify the perceived environmental factors that influence substance use among adolescents living at the U.S.-Mexico border	PhotoVoice 23 youths involved	Informed Consent: All participants made an informed consent Participant Safety and Disclosure: Photographing human subjects, illicit activities, or unsafe spaces was restricted Misrepresentation: Collaboration norms were established to foster respectful discussions and value diverse perspectives. A participatory analytic process was employed, involving youth researchers in the analysis of photographs, narratives, and debrief sessions. Member-checking activities were conducted to validate findings and ensure an accurate representation of the youth researchers' perspectives
3	Smith et al. (2023)	Investigate the complex process of recovery from problem substance use using Photovoice	PhotoVoice 7 service users from a harm reduction drug service	Informed Consent: All participants made an informed consent Privacy and Confidentiality: Confidentiality was discussed with participants and emphasized the importance of being mindful of it when taking photographs of others
4	Kabore et al. (2019)	Assess the risks and protective factors of substance abuse in Ghana, West Africa, using the photovoice method	PhotoVoice 10 participants in recovery from substance abuse and undergoing treatment in the greater Accra region of Ghana	Informed Consent: All participants made an informed consent
5	Drainoni et al. (2019)	Use photography to directly depict the experiences of PWID in the US	PhotoVoice 33 PWID in the US	Informed Consent: All participants made an informed consent Privacy and Confidentiality: The interviews were conducted in private offices or other spaces within community-based organizations
	***Gender identity patients***			
6	Forge et al. (2018)	Explore lived experiences of queer-identified youth of color who are currently experiencing homelessness using Photovoice	PhotoVoice 4 participants from an Atlanta-based service provider for LGBTQ youth	Informed Consent: All participants made an informed consent Privacy and Confidentiality: Participants were guided on uploading photographs to a dedicated email account created specifically for the photovoice project Compensation: The participants were paid $10 remuneration
7	Christensen et al. (2020)	Use existing research to illustrate how using photovoice method for GSIE does involve individuals in exploring gender and sexual identity from multiple levels of social interaction	A conceptual exploration of ways to apply the PV method in the GSIE process	Privacy and Confidentiality: Shared ownership of data was emphasized Power Dynamics: Participants are being made involved in the process of the study as much as possible to diminish the power imbalances between them and the researchers
8	Bauer et al. (2019)	Learn from the unique perspectives of transgender and gender-expansive youth on what may challenge or facilitate gender identity affirmation to improve healthcare usability for those receiving care at a gender affirming clinic	PhotoVoice 16 transgender and gender-expansive youth from a gender clinic at a children’s hospital	Informed Consent: All participants made an informed consent
9	Tan et al. (2021)	Examine the risk and protective factors of cigarette smoking among TGE adults through real-world exemplars	PhotoVoice 47 TGE adults aged ≥ 18 years and currently smoking in the United States	Informed Consent: All participants made an informed consent Privacy and Confidentiality: Participants' information on social media could only be viewed by the participants within their group and the study team
	***People living with HIV***			
10	Lennon-Dearing and Hirschi (2019)	Increase the self-esteem, self-efficacy and decreasing depression among women living with HIV	PhotoVoice 23 women living with HIV	Informed Consent: All participants made an informed consent Privacy and Confidentiality: Participants' photos were displayed via projector and in community exhibits, without clear indications of ongoing privacy protection throughout the study Misrepresentation: The sampling method relied on case managers at local HIV service organizations, potentially introducing bias and limited representativeness Compensation: Participants received digital cameras, photo copies, transportation vouchers, and $20 grocery gift cards per session and exhibit
11	Kimera et al. (2020)	Explore the lived experiences of youth living with HIV/AIDS (YLWHA) with HIV-related stigma in Western Uganda, and to gain insight into how this stigma affects their quality of life	PhotoVoice 11 participants in the age range of 15–19 years from a hospital-based peer support group in Western Uganda (living with HIV/AIDS and had experienced HIV-related stigma)	Informed Consent: All participants, including the caretakers/ parents of the minors, made an informed consent Privacy and Confidentiality: Participants signed photo release forms and were referred to by pseudonyms in recordings, analysis, reporting, and public exhibitions
12	Daniels et al. (2017)	Explore the social factors that influenced HIV care	PhotoVoice 35 MSM with HIV infection who live in townships in Mpumalanga, South Africa	Informed Consent: All participants made an informed consent Privacy and Confidentiality: No facial photos were taken to maintain participant confidentiality; some images were blurred for anonymity
13	Teti et al. (2012)	Explore the ethical opportunities and challenges associated with using photovoice as a community-based participatory research method among women living with HIV/AIDS	PhotoVoice 21 women living with HIV/AIDS recruited from community-based organizations and clinics in two Midwestern cities in the United States	Informed Consent: All participants made an informed consent Participant Safety and Disclosure: Measures were taken to ensure participant safety, including emotional support, creating a safe environment, providing opportunities for self-care, acknowledging the emotional impact of sharing traumatic experiences, and training participants on appropriate photography (to prevent the capture of illegal activities) Privacy and Confidentiality: Measures were taken to protect participants' identities and confidentiality, including the use of pseudonyms, altering identifying details, participant input on the exhibit, and obtaining consent for capturing others in photos Power Dynamics: Researchers involved participants in exhibit planning, acknowledging power imbalances, and giving participants agency in decision-making
14	Lennon-Dearing and Price (2018)	Share stories of the realities of living with HIV through photographic documentation and critical dialogue with peers about the challenges they face, how they have overcome these challenges and what hopes they have for their future	PhotoVoice 23 women living with HIV	Informed Consent: All participants made an informed consent Compensation: Participants were compensated with a $20 Kroger card at each group session for their time
	***Patients living with HIV***			
15	Teti et al. (2012)	Provide a comprehensive overview of Photovoice research among PLWH	Scoping review (22 studies)	Participant Safety and Disclosure: Sharing personal information or photographs related to HIV status may expose participants to risks of stigma and emotional distress. Access to support services is crucial to address potential trauma and provide necessary care Privacy and Confidentiality: Protecting participants' personal stories and photographs is essential to prevent unwanted disclosure and ensure identity protection Power Dynamics: Researchers may exert excessive control over the research process, undervaluing participant input and using them solely for personal gain
16	Witkowski et al. (2021)	Depict the everyday life challenges to be used in several photography exhibits aimed at informing local policy direction	PhotoVoice 9 Latino/a activist	Informed Consent: All participants made an informed consent
17	Lofton et al. (2020)	Develop action plans in Youth Photovoice to address community-level HIV risk in rural Malawi	PhotoVoice 24 youths	Informed Consent: All participants made an informed consent and their consent forms were taken home to their parents for review Participant Safety and Disclosure: The facilitators highlighting the importance of avoiding situations that could threaten their safety and provided access to counseling and support from a psychiatric nurse to address potential abuse or safety concerns Privacy and Confidentiality: Facilitators emphasized the importance of obtaining permission before taking photographs that show identifiable persons
18	Jacobs and Harley (2014)	Introduce and discuss the Photovoice method of data collection in HIV and AIDS-related research	Literature review	Informed Consent: Obtain consent for photographing private spaces, ensuring participants are fully informed, voluntary participation, and have a clear understanding of their rights, with accessible information provided for individuals with cognitive disabilities Participant Safety and Disclosure: Researchers should consider the potential impact of their research on marginalized communities and take steps to minimize harm Privacy and Confidentiality: Researchers should not assume the absence of private spaces in marginalized communities and should respect participants' privacy, avoiding assumptions about personal space or belongings, even in impoverished contexts Misrepresentation: Participants should be involved in the selection of photographs to ensure an accurate representation of their experiences and perspectives
19	Umurungi et al. (2014)	Get the perspectives of these girls on issues of safety and security, particularly in the context of risk of HIV and AIDS	PhotoVoice 16 girls between the ages of 11 and 14 from the province of Ruhengeri	Informed Consent: Obtaining written consent from girls without parents or legal caregivers was complicated. The researchers obtained consent from the Centre administrator and informed the girls about their rights, but some participants were unable to provide written consent due to illiteracy
20	Teti et al. (2013)	Explore how women living with HIV can experience empowerment through Photovoice, a participatory process in which underserved individuals identify, represent, and enhance their lives and communities through photography	PhotoVoice 30 poor and racial/ethnic minority women living with HIV in the United States	Informed Consent: All participants made informed consent Participant Safety and Disclosure: A list of support and counseling resources was provided to ensure participant safety and well-being Privacy and Confidentiality: Pseudonyms were used for participants and identifying information was removed from the data to ensure privacy and confidentiality Power Dynamics: Power differentials based on race/ethnicity, socioeconomic status, and HIV status may have influenced participant willingness to share personal details. The researchers addressed this by actively involving participants in data collection and decision-making, including the choice of photos for publication, to enhance trustworthiness
21	Kennedy et al. (2016)	Identify the effects on women with HIV who chose to show their faces in photographs taken to express their personal experiences	PhotoVoice 35 women with HIV	Informed Consent: The researchers obtained written consent from the participants before starting any project activities Participant Safety and Disclosure: Support resources for HIV-related counseling and assistance were provided. The exhibition of photographs of minors or illegal activities was prohibited Privacy and Confidentiality: Pseudonyms were assigned to participants and all identifying information was removed from the data Power Dynamics: Acknowledging power differentials, the researchers maintained transparency by describing their role in data collection and analysis, considering factors such as race/ethnicity, socioeconomic status, and HIV status
22	Hergenrather et al. (2006)	Identify the influences upon the employment–seeking behavior of PLWHA	PhotoVoice 11 PLWHA who were unemployed and had full-time employment histories after their initial HIV/AIDS diagnosis	Informed Consent: All participants provided informed consent Participant Safety and Disclosure: The researchers provided counseling to participants who required it Compensation: Participants in the study received $10 per session as compensation, which acknowledges their time and contribution to the research
23	Davtyan et al. (2016)	Explore the experiences of African American and Latina/Hispanic women living with HIV-related stigma through PhotoVoice	PhotoVoice 10 African American and Latina/Hispanic women living with HIV	Informed Consent: Participants provided informed consent and signed photo release forms for the publication and exhibition of their photos Privacy and Confidentiality: Pseudonyms were assigned to participants and identifying information from transcripts and photographs was removed Power Dynamics: Power imbalances were acknowledged. Participants were given autonomy in choosing photographs and sharing their own experiences in their own words
	***Sex workers***			
24	Barlow and Hurlock (2013)	Represent through photovoice the lived experience of five exited sex trade workers	PhotoVoice 5 exited sex trade workers as participants	Informed Consent: All participants provided informed consent Privacy and Confidentiality: Participants were given pseudonyms and were allowed to control which photographs were used in public presentations Power Dynamics: Suggested that researchers should be aware of power dynamics between themselves and community members, and should work to ensure that community members have an equal say in decision-making processes Compensation: Participants were reimbursed for babysitting and providing an hourly stipend for participating in meetings
25	Desyllas (2013)	Understand sex workers’ lived experiences through their own artistic self-representation	PhotoVoice Sex workers	Informed Consent: All participants provided informed consent Privacy and Confidentiality: Individual dialogue sessions were conducted with each participant instead of in a group setting Misrepresentation: The researchers used multiple recruitment methods and strategies to reach potential participants Compensation: Participants were compensated with $50 in cash as a thank-you gesture for their time and effort

The studies had varied aims, but a common theme emerged, which was the utilization of photovoice as a method to gain insights into the opinions and experiences of marginalized populations.

### Summary of Ethical Points based on the Articles included in the Review

In photovoice studies, informed consent is identified as the primary ethical concern, as observed in 22 examined papers. These papers highlighted the importance of providing participants with detailed explanations about the project before obtaining their consent (Table 2).

Privacy and confidentiality were emphasized in 18 papers, particularly due to the participation of marginalized populations. Measures such as conducting individual dialogue sessions instead of group settings, using pseudonyms for participants, and ensuring secure handling of recordings and data were mentioned by researchers like Desyllas and Kimera et al.

Participant safety and disclosure were mentioned in 8 papers, addressing concerns about potential restigmatization or retraumatization due to the exposure of sensitive photos. Some studies provided mental health care for participants, prohibited the exhibition of illegal activities, and offered support from professionals like psychiatric nurses, as noted by Kennedy et al. and Lofton et al.

The potential power imbalance between researchers and marginalized participants was discussed in 7 papers. Strategies to address this issue included involving participants in the study process as much as possible to diminish power imbalances, as mentioned by Christensen et al. Teti et al. acknowledged that power imbalances could arise from factors such as race/ethnicity, socioeconomic status, and HIV status.

Compensation for participants was mentioned in 6 papers, with examples such as providing monetary compensation, vouchers, or other forms of recognition for their contributions, as seen in Barlow et al.

Four papers addressed the potential misrepresentation of participants' intentions through the selected photographs. To prevent misrepresentation, involving participants in the selection of photographs was suggested by Jacobs et al. Additionally, the potential bias introduced by sampling methods and its impact on misrepresentation was discussed by Lennon-Dearing et al.

**Table 2:** Summary of Ethical Issues

Ethical issues	Article	Descriptions
Informed Consent	(2), (3), (4), (5), (6), (8), (9), (10), (11), (12), (13), (14), (16), (17), (18), (19), (20), (21), (22), (23), (24), (25)	• Obtaining informed consent from participants • Differentiation between consent processes; engaging participants in education and discussion related to photography ethics; providing written material and instructions; reviewing consent for publication of pictures once they have been developed, and providing a copy of pictures to participants • Everyone in the pictures should provide informed consent
Participant Safety and Disclosure	(2), (13), (15), (17), (18), (20), (21), (22)	• Ensuring participant safety and disclosure decisions • Taking pictures of others without permission or pictures that risk portrayal of an individual, community, or group in a negative light • Re-stigmatisation when conducting Photovoice with individuals who are experiencing illness and disability • Photos are being disclosed for commercial purposes • Exposure of illegal activities
Privacy and Confidentiality	(1), (3), (5), (6), (7), (9), (10), (11), (12), (13), (15), (17), (18), (20), (21), (23), (24), (25)	• Privacy concerns about whether social media data should be considered public or private • Ownership • Methods of disseminating and discussing the photos captured • Privacy of identity of those being captured in the photos
Misrepresentation	(2), (10), (18), (25)	• The challenge of interpreting photographs and ensuring that their voices are accurately represented
Power Dynamics	(7), (13), (15), (20), (21), (23), (24)	• Power dynamics between researchers and participants • Respect participants' autonomy
Compensation	(6), (10), (14), (22), (24), (25)	• Compensating participants for their time and effort in contributing to the research project

(1) Barlow and Hurlock (2013); (2) Bauer et al. (2019); (3) Christensen et al. (2020); (4) Daniels et al. (2017); (5) Davtyan et al. (2016); (6) Desyllas (2013); (7) Drainoni et al. (2019); (8) Forge et al. (2018); (9) Hergenrather et al. (2006); (10) Jacobs and Harley (2014); (11) Kabore et al. (2019); (12) Kennedy et al. (2016); (13) Kimera et al. (2020); (14) Lennon-Dearing and Hirschi (2019); (15) Lennon-Dearing and Price (2018); (16) Lofton et al. (2020); (17) Padilla et al. (2018); (18) Salerno Valdez et al. (2019); (19) Smith et al. (2023); (20) Tan et al. (2021); (21) Teti et al. (2018); (22) Teti et al. (2012); (23) Teti et al. (2013) (24) Umurungi et al. (2014); (25) Witkowski et al. (2021).

## Discussion

Ethical considerations are frequently discussed in the literature on photovoice projects, particularly because these projects often focus on marginalized populations whose rights are frequently overlooked and undervalued (Wang and Redwood-Jones 2001). Upholding ethical standards is crucial in order to prevent the exploitation of these populations.

In our review of 25 papers, which focused specifically on marginalized populations such as drug users, gender identity patients, people living with HIV (PLWH), patients living with HIV, and sex workers, we have identified and categorized ethical concerns and challenges into six main groups: Informed Consent, Participant Safety and Disclosure, Privacy and Confidentiality, Misrepresentation, Power Dynamics, and Compensation. Additionally, some papers offer suggestions to address and overcome these ethical problems, which are grouped accordingly within each respective category.

In our review of 25 papers, the most prevalent ethical concern was obtaining informed consent. It is important to ensure that participants have a comprehensive understanding of their rights and the project before providing their consent. However, obtaining consent can be more complex in certain circumstances, such as when it involves minors, illiterate individuals, or those without legal guardians. For instance, Kimera et al. (2020) conducted a photovoice project involving participants aged 15–19. In addition to obtaining consent from the participants themselves, they also obtained informed consent from the caretakers or parents of the minors. Similarly, Lofton et al. (2020), who conducted a photovoice project with 24 youths, required participants to have their parents review the consent forms before providing their own consent. Special consideration was given to individuals with cognitive disabilities in obtaining consent, as highlighted by Jacobs and Harley (2014). They emphasized the importance of providing accessible information and ensuring understanding for this particular group. Whereas, Umurungi et al.'s photovoice project (2014) encountered difficulties in obtaining written consent from girls without parents or legal caregivers. As a resolution, the researchers obtained consent from the Centre administrator after informing the girls about their rights. Nonetheless, due to illiteracy, some participants could not provide written consent. Overall, the process of obtaining informed consent requires careful attention and tailored approaches to address the specific needs and circumstances of the participants involved.

Participant Safety and Disclosure is of utmost importance in photovoice projects, as it entails protecting participants from stigma, re-traumatization, and potential harm while empowering them to maintain control over their images and stories. For example, a study implemented restrictions on photographing illicit activities or unsafe spaces (Salerno Valdez et al. 2019), Another study by Teti et al. (2012, 2018) provided guidance on appropriate photography to avoid capturing illegal activities, and Kennedy et al. (2016) prohibited the exhibition of photographs involving minors or illegal activities. Additionally, Lofton et al. (2020) prioritized emotional support by including a psychiatric nurse in their team to ensure participant well-being.

Privacy and Confidentiality emerges as significant ethical concerns in our review, following Informed Consent. Protecting participants' privacy was crucial to ensure their safety, as mentioned by Smith et al. (2023) who taught participants about confidentiality when photographing others. Measures such as conducting interviews in enclosed spaces, as done by Drainoni et al. (2019), and opting for individual dialogue sessions instead of group settings, as practiced by Desyllas (2013), further safeguarded privacy. Forge et al. (2018) guided participants on securely uploading photographs to a dedicated email account, while Christensen et al. (2020) emphasized shared ownership of data with participants. However, few papers addressed privacy during data collection and dissemination, as seen in Lennon-Dearing and Hirschi (2019) where confidentiality measures during photo display were unclear. To protect the confidentiality, pseudonyms were used in recordings, analysis, reporting, and public exhibitions, and identifiable features were blurred, as demonstrated by some studies (Daniels et al. 2017; Kimera et al. 2020). Additionally, respecting the privacy of those being photographed, not just the participants, was highlighted by Jacobs and Harley (2014), urging researchers to avoid assumptions about personal space or belongings in marginalized communities, even in impoverished contexts.

Misrepresentation was a notable concern addressed in the studies, particularly in relation to the sampling method used to recruit participants. There was a study that recruited their participants solely through case managers at local HIV service organizations, which may have introduced a potential source of bias and misrepresentation (Lennon-Dearing and Hirschi 2019). Desyllas (2013) also highlighted the importance of employing multiple recruitment methods to mitigate this issue. To address misrepresentation, Salerno Valdez et al. (2019) conducted member-checking activities to validate findings and ensure an accurate representation of the youth researchers' perspectives. Jacobs and Harley (2014) emphasized the involvement of participants in the photography selection process as another means to prevent misrepresentation.

Moreover, Power Dynamics was also a significant ethical concern in photovoice projects, particularly due to the involvement of marginalized populations and the potential for researchers to hold a position of perceived superiority. To address this, strategies were implemented, such as involving participants in the research process to ensure their equal participation and decision-making, as advocated by Christensen et al. (2020) and Barlow and Hurlock (2013). Teti et al. (2012, 2018) highlighted the risk of researchers exerting excessive control and undervaluing participant input for personal gain, while also acknowledging the influence of power differentials based on race/ethnicity, socioeconomic status, and HIV status. Maintaining transparency about the researchers' roles in data collection and analysis, as demonstrated by Kennedy et al. (2016) and their team, aimed to mitigate power imbalances.

Compensation for participants was discussed in multiple studies, acknowledging the importance of valuing their time and contributions. It was emphasized to strike an appropriate balance in compensation, ensuring it was not perceived as a transactional exchange, but rather as a gesture of appreciation for their involvement. Monetary compensation was provided in studies by Forge et al. (2018), Hergenrather et al. (2006), Barlow and Hurlock (2013), and Desyllas (2013). Lennon-Dearing and Hirschi (2019) went beyond monetary compensation by offering additional benefits such as digital cameras, photo copies, transportation vouchers, and grocery gift cards to acknowledge participants' efforts during sessions and exhibits.

The strength of this paper lies in its rigorous review process involving two independent reviewers, which effectively minimizes biases. Furthermore, it is among the pioneering papers investigating the ethical considerations surrounding photovoice. Additionally, the paper employs a robust scoping review methodology, ensuring a comprehensive synthesis of existing literature by incorporating all relevant sources. It is worth noting that the paper includes a summary of findings derived from the existing literature, offering valuable insights and recommendations for future photovoice studies.

One potential weakness of this paper is that the search terms used may not have been exhaustive, which could have resulted in the researchers overlooking potentially relevant articles. Additionally, the inclusion of only English databases might introduce bias by excluding relevant studies published in other languages. Furthermore, the exclusion of grey literature may limit the comprehensiveness of the review. Moreover, the paper's narrow focus on photovoice research projects involving marginalized communities and key populations might restrict the generalizability of the findings to other types of research projects or different populations.

## Conclusion

Lastly, the ethical landscape of photovoice studies with marginalized populations highlights the importance of prioritizing participant rights, well-being, and empowerment. This study reinforces the significance of addressing key ethical concerns, including informed consent, participant safety and disclosure, privacy and confidentiality, misrepresentation, power dynamics, and compensation. Integrating technology can enhance the informed consent process by providing secure electronic platforms for participants to provide their consent, ensuring privacy and confidentiality. Additionally, technological tools can facilitate secure data storage and transmission, safeguarding participant safety and confidentiality. Fostering collaborative decision-making processes is crucial to mitigate power imbalances. Engaging participants as active partners in the research process not only empowers them but also reduces the risk of misrepresentation. This participatory approach promotes authenticity and inclusivity. Regarding compensation, researchers should explore alternative forms beyond monetary rewards. This can include providing access to resources, services, or opportunities that address participants' needs, thereby fostering a reciprocal relationship. By adopting these innovative approaches, researchers can navigate the ethical complexities of photovoice studies.
